# Efficient clonal seeds sorting for apomictic hybrid rice using a pollen‐specific gene switch system

**DOI:** 10.1111/pbi.70031

**Published:** 2025-03-19

**Authors:** Yijie Zhan, Yumei Xia, Yao Wang, Siqing Liu, XiuLi Zhang, Shuo Xiong, Qiming Lv, Mengliang Cao

**Affiliations:** ^1^ Long Ping Branch, College of Biology Hunan University Changsha China; ^2^ State Key Laboratory of Hybrid Rice Hunan Hybrid Rice Research Center Changsha China; ^3^ National Center of Technology Innovation for Saline‐Alkali Tolerant Rice in Sanya Sanya China; ^4^ School of Breeding and Multiplication (Sanya Institute of Breeding and Multiplication) Hainan University Sanya China

**Keywords:** *Cre/Loxp + FRT*, specific promoter, apomixis, fluorescence sorting, site‐specific recombination

## Abstract

Significant progress in apomictic hybrid rice development faces challenges like achieving high induction rates and seed‐setting efficiencies, and distinguishing clonal from zygotic embryos. To address the challenge of selecting clonal seeds, we developed a dual‐fluorescence labelling gene switch system using the recombinase *Cre/LoxP + FRT*. Initially, this system was tested in callus tissue under a constitutive promoter; then, we replaced the promoter with a pollen‐specific one to develop the pollen‐specific gene switch (PSGS) system. The effectiveness of PSGS in rice pollen was subsequently validated. After confirming its functionality, we co‐transformed the PSGS vectors with apomixis vectors in hybrid rice Yongyou 2640 (YE) and Yongyou 4949 (YS) using *Agrobacterium*‐mediated transformation. Finally, we identified 18 *MiMe* mutants carrying the PSGS; the progeny of 16 lines were all red fluorescence seeds (zygotic embryo). Surprisingly, line L47‐4 and L151‐1 yielded 418 (*n* = 418) and 218 (*n* = 1279) non‐fluorescent seeds in the T_1_ generation, respectively. The ploidy detection of non‐fluorescent seeds showed that 57 (*n* = 68) and 64 (*n* = 72) were diploid in Line L47‐4 and L151‐1, individually. This phenomenon was reproducible in the T_2_ generation; 97 (*n* = 121) and 164 (*n* = 187) non‐fluorescent seeds were diploid from line L47‐4 and L151‐1, respectively. This study demonstrates the ability of PSGS to distinguish between clonal seeds and zygotic seeds, with a sorting accuracy rate ranging from 80.2% to 88.9%, which is essential for improving clonal seed purity and advancing apomixis in rice cultivation.

## Introduction

In the 1970s, academician Yuan successfully developed the three‐line hybrid rice, which significantly increased rice yields per unit area in China. He envisioned a strategic evolution in rice breeding, progressing from the three‐line to the two‐line system and ultimately to the one‐line system (Yuan, 1987). In recent years, synthetic apomixis has developed rapidly, and achieving apomixis is also hailed as the ‘Holy Grail of Agriculture’. The *BBM1* gene, a member of the *AP2* family, is ectopically expressed under the control of the oocyte‐specific promoter *pAtDD45* (Khanday *et al*., 2019). In conjunction with the Mitosis instead of Meiosis (MiMe) approach, this leads to clonal seeds induction rates ranging from 11.1% to 29.2% (Khanday *et al*., 2023). By mutating the genes *pair1*/*osrec8*/*ososd1*/*osmatl*, synthetic apomixis was successfully induced in rice, with induction rates varying from 4.7% to 9.5% and seed‐setting rates from 3.7% to 5.2% (Wang *et al*., 2019). Subsequent research combined the *MiMe* technique with the use of promoters *pAtECS* and *pOsECS* to ectopically express *BBM1*, achieving a parthenogenesis frequency exceeding 95%, although the seed‐setting rate ranged between 27% and 35.5% (Vernet *et al*., 2022). Additionally, the oocyte‐specific promoter *pAtDD45* was employed to ectopically express *BBM4*, which could attain a maximum seed‐setting rate of 82.6%, but at the expense of a reduced induction rate, falling below 3% (Wei *et al*., 2023). Similarly, using the oocyte‐specific promoter *pAtDD45* to ectopically express the *WUS* gene also resulted in lines with seed‐setting rates comparable to the control, but the induction rate ranged from 0.5% to 21.7% (Huang *et al*., 2024). Despite substantial theoretical and practical advancements in the one‐line method of hybrid rice in recent years, synthetic apomixis technology continues to confront challenges, including achieving high induction and seed‐setting rates simultaneously and distinguishing between clonal and zygotic embryos.

Gene switch systems commonly employed in rice include the *Cre/Loxp* system and the *FLP/FRT* system (Srivastava and Nicholson, 2006; Nandy and Srivastava, 2011). The *Cre/Loxp* system, a site‐specific recombinase technology, enables genetic editing operations, such as deletion, insertion, translocation and inversion at defined DNA loci (Rao *et al*., 2010). This system is applicable in specific cell types or in response to external stimuli, allowing for precise modification of cellular DNA. The *Cre* recombinase recognizes inverted repeat sequences adjacent to *Loxp* sites, forms dimers and then dimerizes with another at a *Loxp* site to form a tetramer, which cleaves the DNA sequence between the two sites (Stachowski *et al*., 2022). The *FLP/FRT* system operates similarly to the *Cre/Loxp* system, facilitating gene deletion or insertion by targeting and cleaving the DNA segment flanked by two *FRT* sites (Luo and Kausch, 2002a, 2002b). In various experimental contexts, the deletion efficiency of the *Cre/Loxp* system ranges from 6.8% to 83.3%, whereas the efficiency of the *FLP/FRT* system varies from 55.0% to 86.5% (Hu, 2013; Nguyen *et al*., 2014; Wen, 2015; Zhao *et al*., 2011). However, there are few comparative studies evaluating the deletion efficiencies of these two systems.

To enhance deletion efficiency, we developed two deletion systems utilizing the fusion recognition sites *Loxp + FRT* (Luo *et al*., 2007): *Cre/Loxp* + *FRT* and *FLP/Loxp* + *FRT*. The study reveals that the deletion efficiency is relatively low when the *Cre/Loxp* or *FLP/Loxp* system is used in tobacco pollen and seeds, with none of the transgenic lines achieving 100% deletion efficiency. Nonetheless, the employment of the fusion recognition sites *Loxp* + *FRT* significantly enhances deletion efficiency with the single expression of either *Cre* or *FLP*, and multiple transgenic lines have achieved 100% deletion efficiency.

This study introduces a gene switch system for the one‐line method of hybrid rice, designed to facilitate the distinction between cloned and zygotic embryos by effectively screening for apomictic hybrid rice using fluorescent markers. We bombarded deletion system vectors carrying plasmid microparticles into rice callus tissue and compared the deletion efficiency of the *Cre/Loxp + FRT* and *FLP/Loxp + FRT* systems through fluorescence observation. Subsequently, we developed a pollen‐specific gene switch (PSGS) system by substituting the constitutive promoter with a pollen‐specific promoter. This development, in conjunction with apomictic vectors created in our laboratory, enabled the efficient sorting of apomictic and zygotic embryos. The methodology was validated using the hybrid rice combinations Yongyou 2640 and Yongyou 4949, demonstrating the high sorting efficiency of the PSGS across generations. This research not only addresses critical challenges in the production of apomictic rice but also paves the way for future applications of gene switch systems in agricultural biotechnology.

## Results

### The *Cre/Loxp* system demonstrated superior deletion efficiency in rice callus

**Figure 1:**
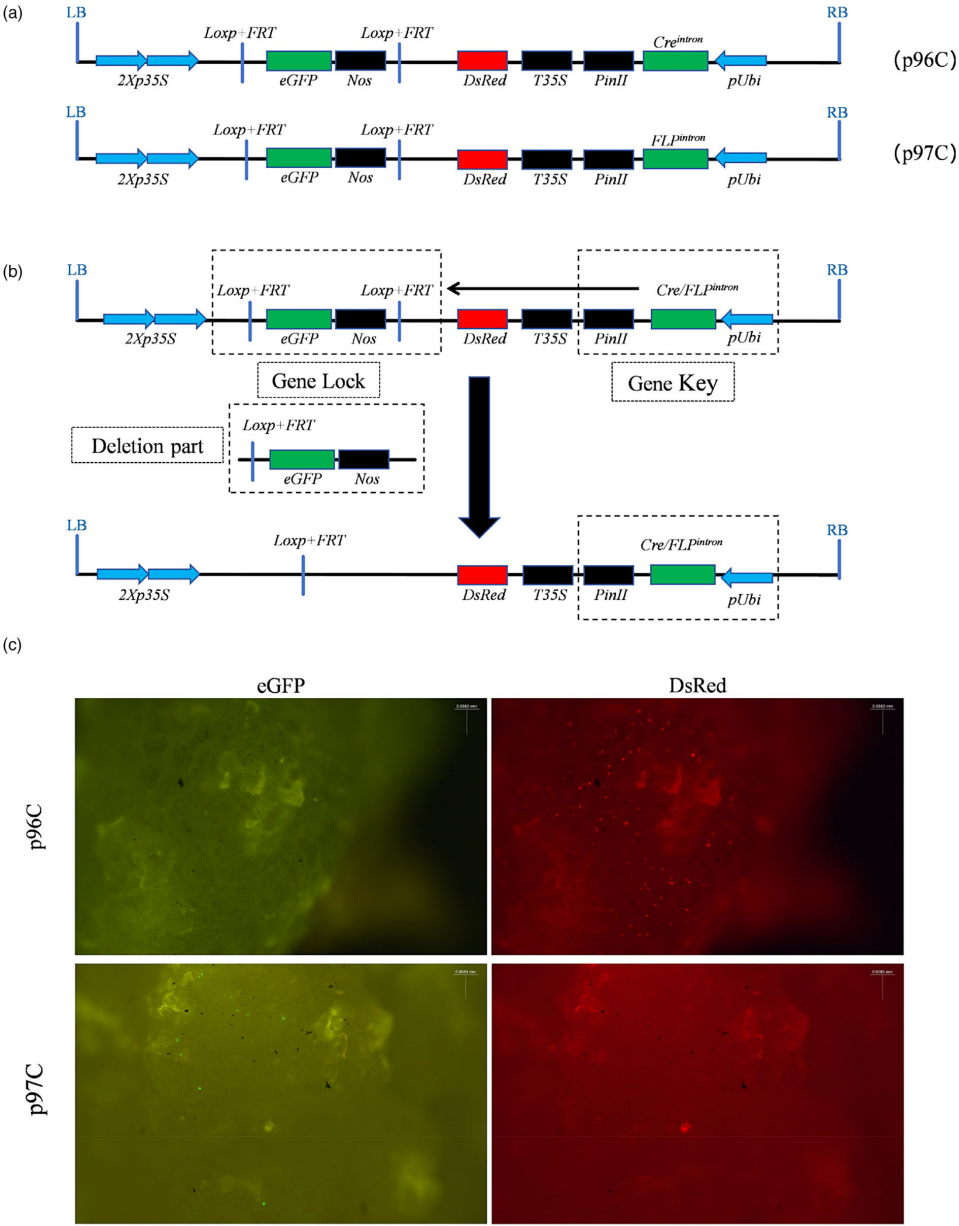
Schematic representation of p96C and p97C vector constructs, deletion mechanism, and fluorescence expression patterns. (a) The structural diagram of the T‐DNA region for constructing a gene deletion system, utilizing promoters to regulate gene expression and induce specific fluorescence signals. Upper panel: p96C; Lower panel: p97C; LB and RB: left and right borders of the T‐DNA; *p35S*: Cauliflower Mosaic Virus (CaMV) promoter; *Loxp + FRT*: recombinase‐specific recognition site; *eGFP*: green fluorescent protein gene; *Nos*: terminator; *DsRed*: red fluorescent protein gene; *T35S*: terminator of *p35S*; *PinII*: terminator; *Cre* ^*intron*^/*FLP* ^*intron*^: both *Cre* and *FLP* are recombinase genes, with *Cre* derived from P1 phage and *FLP* from yeast, both containing a 190 bp intron fragment; *pUbi*: ubiquitin gene promoter. (b) Illustration of the deletion system mechanism. (c) Fluorescence expression patterns for p96C and p97C. Scale bars, 0.0583 mm.

In the expression vectors p96C and p97C (Figure 1a), the *Loxp + FRT*‐*eGFP*‐*Nos*‐*Loxp + FRT* cassette acts as the ‘gene lock’ while the *pUbi*‐*Cre* ^*intron*^‐*PinII* cassette functions as the ‘gene key’ Upon specific recognition of the *Loxp + FRT* site by the ‘gene key’ and subsequent deletion of the ‘gene lock’ red fluorescence is detected; in contrast, the absence of deletion results in the observation of green fluorescence (Figure 1b).

The plasmids p96C and p97C were successfully delivered into callus tissue via biolistic bombardment at concentrations of 2 and 5 ng, respectively. The transient fluorescence expression was monitored, and the deletion efficiency of the two recombinases was statistically assessed the day after bombardment. Under the stereomicroscope, both red and green fluorescent signals were visible in the bombarded callus. In the case of p96C, the number of red fluorescent spots significantly outnumbered the green spots, while in p97C, the counts of red and green fluorescent spots were approximately equal (Figure 1c). Statistical analysis showed that at a plasmid concentration of 2 ng, p96C displayed 122 red and 27 green fluorescent spots, yielding a relative deletion efficiency of 81.9%, whereas p97C showed 49 red and 36 green fluorescent spots, with a relative deletion efficiency of 57.6% (Table S1). At a 5 ng plasmid concentration, p96C had 452 red and 48 green fluorescent spots, resulting in a relative deletion efficiency of 90.4%, whereas p97C had 57 red and 69 green fluorescent spots, corresponding to a relative deletion efficiency of 45.2% (Table S1). These data suggest that the *Cre/Loxp + FRT* system exhibits a markedly higher deletion efficiency in rice callus compared with the *FLP/Loxp + FRT* system.

### PSGS was verified in rice pollen

**Figure 2:**
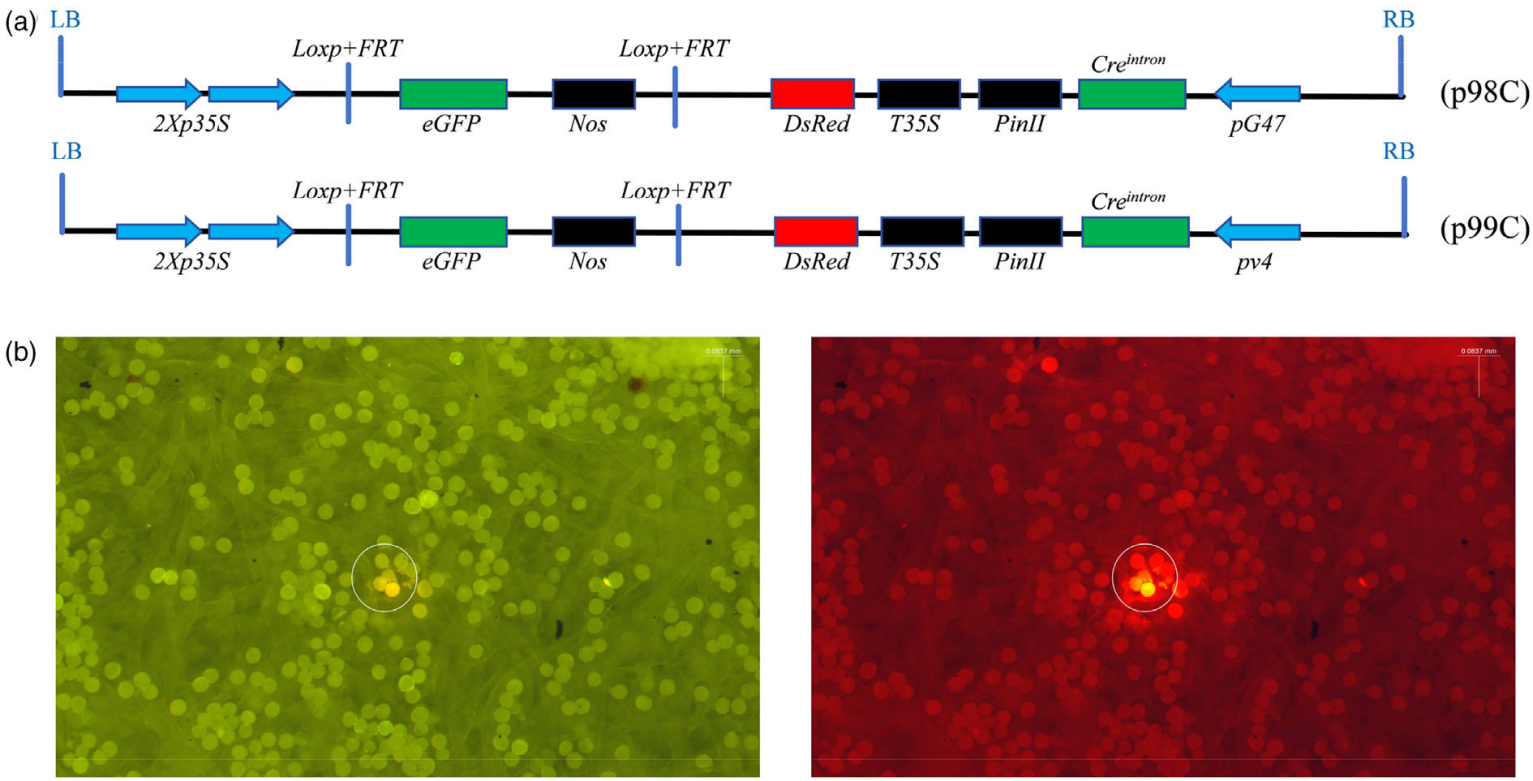
Validation of pollen‐specific promoter activity (a) The T‐DNA structure diagram depicts the molecular elements incorporated into the PSGS; *pG47*: a maize pollen development‐specific promoter; *pv4*: a rice anther‐specific promoter. (b) Fluorescence microscopy images of pollen grains show red fluorescence, where a white circle is used to delineate the area of fluorescence. Scale bars, 0.0873 mm.

The p96C vector was modified by replacing the constitutive promoter *pUbi* with the pollen‐specific promoter *pG47*, leading to the creation of the vector p98C (Figure 2a). Similarly, the pollen‐specific promoter *pv4* was integrated into p96C in place of *pUbi*, which led to the development of the vector p99C (Figure 2a). Following the biolistic delivery of rice pollen with microspheres coated with the p98C and p99C plasmids, we detected the expression of the red fluorescent protein in the pollen grains, thereby validating the functionality of the pollen‐specific gene switch system (Figure 2b).

### PSGS one‐line hybrid rice was produced

**Figure 3:**
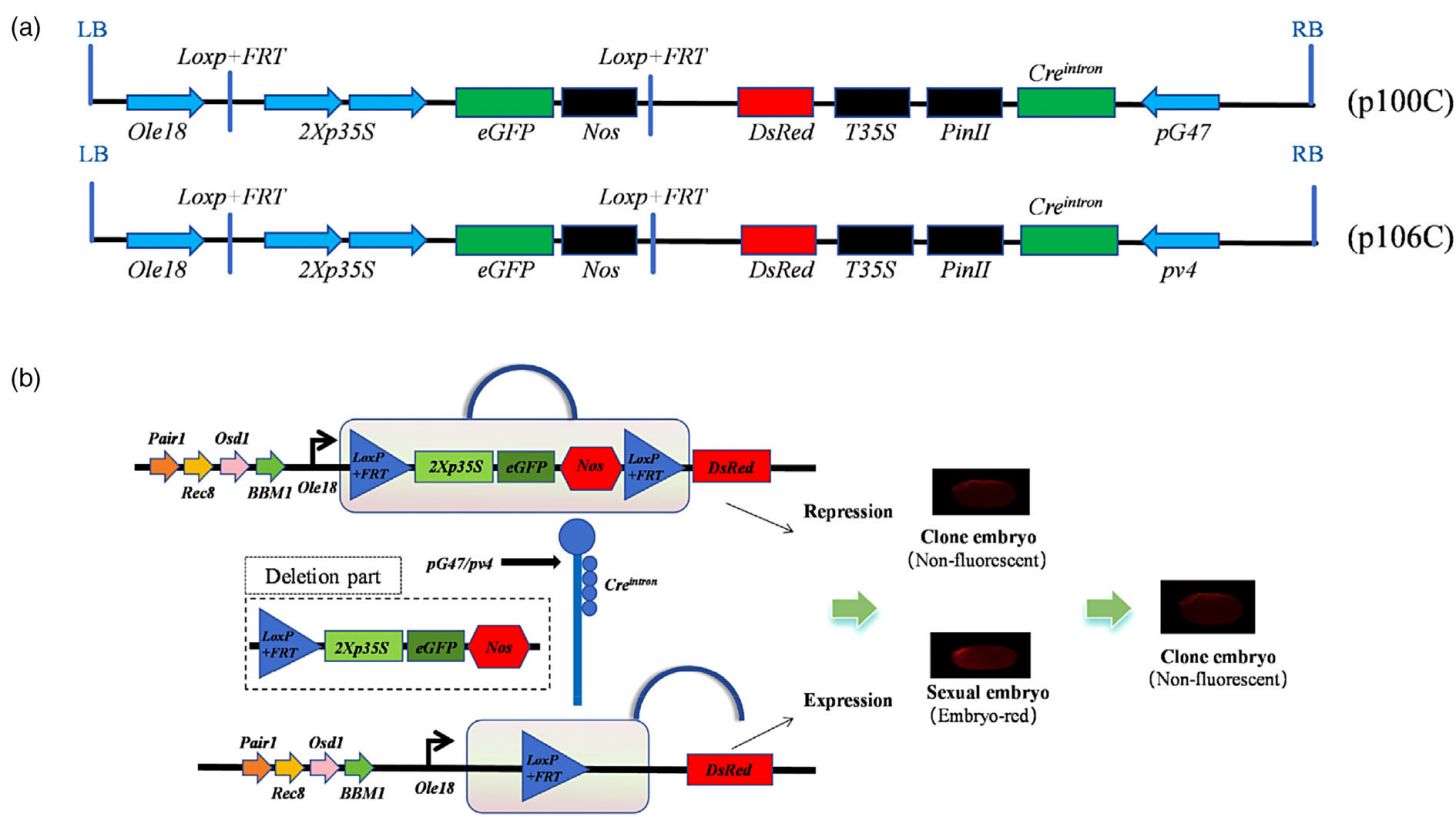
Overview of the pollen‐specific gene switch system (PSGS) and its sorting principle. (a) The T‐DNA structure diagram depicts the molecular elements incorporated into the PSGS; *Ole18*: rice embryo‐specific promoter. (b) Sorting mechanism of the PSGS.

Building on the vectors p98C and p99C, we implemented a modification by replacing the *2Xp35S*‐*Loxp + FRT* sequence with *Ole18*‐*Loxp + FRT*‐*2Xp35S*, resulting in the new vectors p100C and p106C, respectively (Figure 3a). In this PSGS, the pollen‐specific promoters *pG47* and *pv4* drive the expression of the recombinase *Cre* ^*intron*^ within pollen grains. This recombinase is designed to target and excise sequences flanked by the *Loxp + FRT* fusion sites. After pollination and fertilization, seeds that have experienced the deletion event express red fluorescence in the embryo, which is controlled by the embryo‐specific promoter. Conversely, embryos resulting from apomixis are not subject to pollination, fertilization or the deletion process, and as a result, they do not exhibit red fluorescence. Through fluorescence detection, the targeted apomictic seeds can be selectively pinpointed; hence, streamlining the seed sorting process (Figure 3b).

The vectors p100C and p106C were co‐transformed with the apomixis vectors *‘sgMiMe’_pAtDD45:BBM1* (p94C) and *‘sgMiMe’_pAtMYB98 + pAtDD1 + pOsECA1‐like1:WUS_pAtDD45:BBM1* (p95C), which were previously developed in our laboratory (Dan *et al*., 2024), via *Agrobacterium*‐mediated transformation in the YE and YS genetic backgrounds. The transformation process resulted in the generation of transgenic plants for the following combinations: 56 plants for p100C‐p94C, 65 plants for p100C‐p95C, 64 plants for p106C‐p94C and 57 plants for p106C‐p95C. From these, 11, 3, 2 and 2 *MiMe* mutants were obtained, respectively, with co‐transformation efficiencies of 19.6%, 4.6%, 3.1% and 3.5% (Table S2). Field observations of fluorescence were conducted for the 18 mutant lines. Two lines, L47‐4 and L151‐1, which exhibited the desired fluorescence characteristics, were identified from the p100C‐p94C (transformed YE) and p106C‐p95C (transformed YE) combinations, respectively. Furthermore, line L39‐1, derived from the p100C‐p94C transformed YE background, exhibits robust fluorescence that does not align with the specified fluorescence criteria; however, it retains significant research merit. Sequence analysis of the gene mutation sites in L47‐4, L151‐1 and L39‐1 revealed that all three lines carried a homozygous mutations in *OSD1*, *PAIR1* and *REC8* (Figure S1).

### Clonal seeds were isolated efficiently by using the PSGS

**Figure 4:**
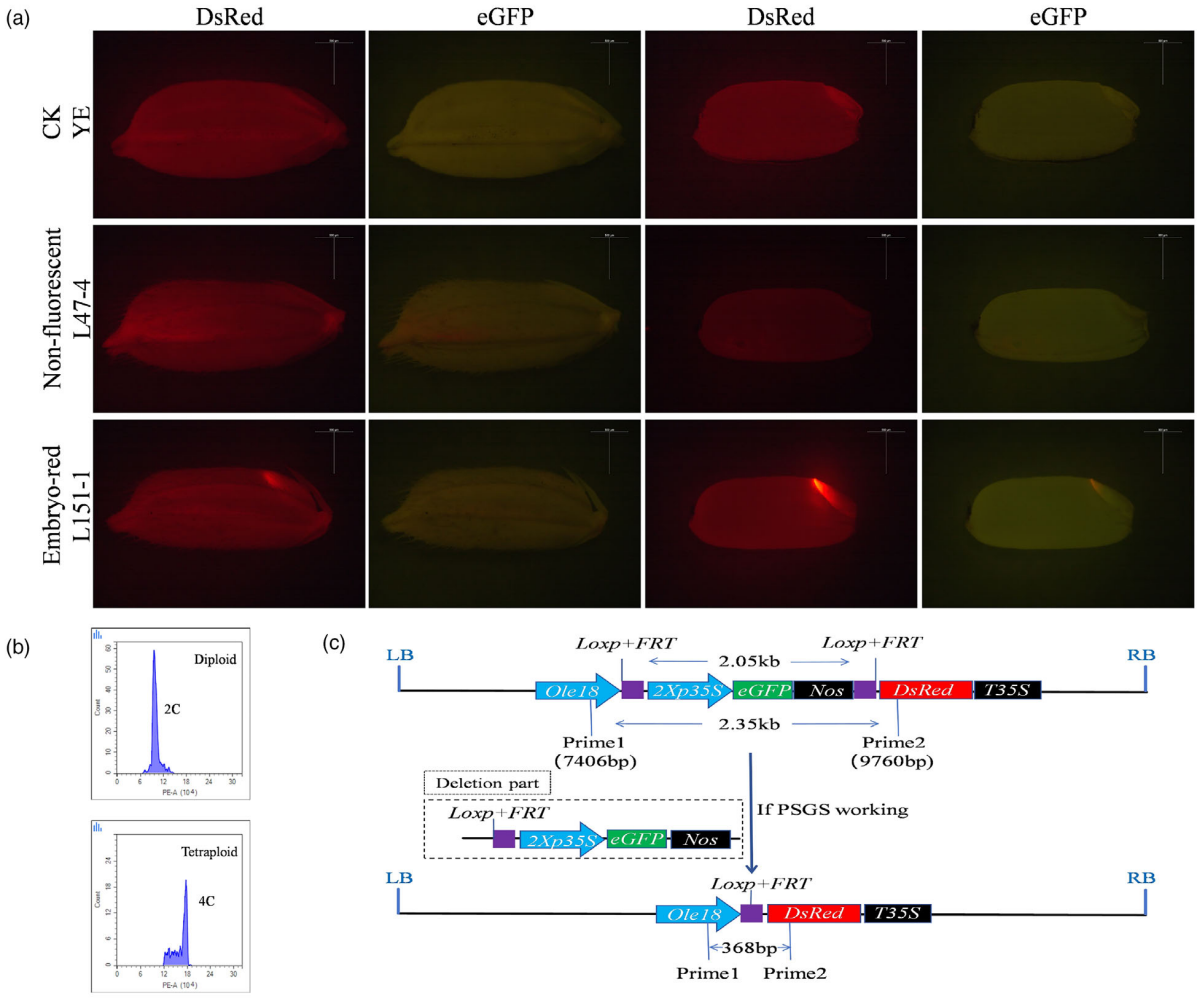
Identification of seeds using the pollen‐specific gene switch (PSGS) with a one‐line method and the principle of gene deletion. (a) Stereomicroscopic images showing wild‐type seeds, non‐fluorescent L47‐4 seeds and L151‐1 seeds with red embryo fluorescence under red and green fluorescence illumination, along with the fluorescence of dehusked seeds. Scale bars, 500 μm. (b) Representative flow cytometry histograms depicting DAPI‐stained nuclear suspensions from the leaves of diploid (left) and tetraploid (right) progeny. (c) Schematic representation of the sizes of deletion fragments associated with the PSGS.

In the T_1_ generation of L47‐4, 418 seeds were collected, all of which lacked red fluorescence in the embryos, classified as ‘non‐fluorescent’ (Figure 4a). The non‐fluorescent seeds constituted 100% of the total (Table S3). For the T_1_ generation of L151‐1, 1279 seeds were harvested, with 218 being non‐fluorescent, and the rest displaying red fluorescence in the embryos, termed ‘embryo‐red’ (Figure 4a). The non‐fluorescent seeds represented 17.0% of the total (Table S3). In the T_2_ generation of the L47‐4 line, red fluorescence was observed in the seed embryos, with only 2.8% of the seeds being non‐fluorescent, while the majority exhibited embryo‐red fluorescence (*n* = 6680, Table S3). For the T_2_ generation of L151‐1, 10.4% of the seeds were non‐fluorescent, and the rest showed embryo‐red fluorescence (*n* = 9788, Table S3).

From the L47‐4 and L151‐1T_1_ generations, we selected 68 and 72 non‐fluorescent germinated seeds for testing, resulting in the cultivation of 57 and 64 diploid plants, respectively, with the rest being tetraploid (Table S4). The sorting accuracy rates were 83.8% and 88.9%, respectively. From the T_2_ generations of L47‐4 and L151‐1, we selected 121 and 187 germinated non‐fluorescent seeds for testing, which resulted in the cultivation of 97 and 164 diploid plants, respectively, with the rest being tetraploid (Table S4). The sorting accuracy rates were 80.2% and 87.7%, respectively. We also cultivated the sorted embryo‐red seeds. In the T_1_ generation of embryo‐red seeds from L47‐4 and L151‐1, no red fluorescent seeds were observed in L47‐4; hence, it was not tested, whereas the diploid rate for L151‐1 was 10.4% (7/67, Table S4). In the T_2_ generation of embryo‐red seeds from L47‐4 and L151‐1, the diploid rates were 5.3% (7/126) and 11.2% (19/150), respectively (Table S4).

In the T_1_ generation, the proportion of clonal seeds for L47‐4 and L151‐1 before sorting was 83.8% (57/68, unsorted) and 28.6% (22/77), respectively. After sorting, the proportion of clonal seeds in the non‐fluorescent seeds was 83.8% and 88.9%, respectively, with an increase of 60.3% in the proportion of clonal seeds for L151‐1. In the T_2_ generation, the proportion of clonal seeds for L47‐4 and L151‐1 before sorting was 9.7% (13/134) and 27.0% (47/174), respectively. After sorting, the proportion of clonal seeds in the non‐fluorescent seeds was 80.2% and 87.7%, respectively, with increases of 70.5% and 60.7% in the proportion of clonal seeds.

To confirm the deletion site and the effectiveness of the PSGS system, PCR amplification and sequencing were conducted on the leaf DNA of T_1_ generation plants from lines L47‐4 and L151‐1. The *Loxp* + *FRT* fusion recognition sites were positioned at nucleotide positions 7536–7621 and 9523–9608. Specific primers are designed to anneal to the regions flanking the *Loxp* + *FRT* fusion recognition site. In the absence of deletion, PCR amplification produced a 2.35 kb fragment; conversely, if a deletion occurs, a 368 bp fragment is amplified (Figure 4c). In diploid samples from the non‐fluorescent groups of L47‐4 and L151‐1, only the 2.35 kb fragment was observed (*n* = 23, Figure S2). Meanwhile, tetraploid samples from the embryo red groups of L47‐4 and L151‐1 displayed both the 2.35 kb and 368 bp fragments (*n* = 23, Figure S2). Sequencing of the amplified fragments verified that the 2.35 kb fragment contained the entire ‘gene lock’ sequence. In contrast, the 368 bp fragment lacks the sequence between the fusion recognition sites, suggesting that only one fusion recognition site is retained after the deletion event (Figure S3).

The T_1_ generation of L47‐4 and L151‐1 lines was evaluated for agronomic traits in a sample of 8 randomly selected plants, with the results subject to statistical analysis (Figure S4). The average plant heights for L47‐4 and L151‐1 were 122.7 and 120.7 cm, respectively, and their average panicle lengths were 23.4 and 19.3 cm, respectively. In comparison, the wild‐type plants exhibited an average height of 117.7 cm and an average panicle length of 22.8 cm. The seed‐setting rates for L47‐4 and L151‐1 were 48.9% and 55.5%, respectively, while the wild‐type plants had a seed‐setting rate of 88.6%.

## Discussions

This study successfully demonstrates the sorting capability of the PSGS system in synthetic apomixis rice. Utilizing the *Cre/Loxp* recombinase system (Chen *et al*., 2017), we developed a PSGS that enables the categorization of seeds into two types based on fluorescence: zygotic embryo seeds with red fluorescence and clonal embryo seeds without fluorescence. This distinct fluorescence allows for straightforward sorting. After sorting, the non‐fluorescent seeds were planted, and both field phenotype observations and flow cytometry data confirmed the effective selection of diploid plants, with a sorting accuracy rate ranging from 80.2% to 88.9%. Molecular detection aligned with the sorting results, as all diploid plants (non‐fluorescent) displayed undeleted bands, indicating clonality and apomictic origin.

Previous studies have struggled to achieve high induction and seed‐setting rates simultaneously in apomictic lines; lines with higher seed‐setting rates often have lower induction rates (Dan *et al*., 2024; Huang *et al*., 2024; Khanday *et al*., 2023; Song *et al*., 2024; Vernet *et al*., 2022; Wang *et al*., 2019; Wei *et al*., 2023). However, by integrating the PSGS with lines that have high seed‐setting rates but low induction rates, it is possible to isolate clonal seeds with non‐fluorescent embryos through fluorescence‐assisted sorting. The combination of the PSGS with the one‐line method significantly improves seed purity and accelerates the adoption of parthenogenetic rice cultivation. This integrated approach ensures genetic uniformity in the produced seeds, which is crucial for maintaining the traits of parthenogenetic rice, and it streamlines the application of this technology in agricultural settings.

Additionally, the majority of apomictic generations are stably inherited. Nonetheless, some cases of unstable inheritance have been documented. For instance, a study involving the naturally occurring apomictic plant *Hieracium pilosella* cultivated 11 hybrid genotypes (lines). Within these hybrid lines and a parental line, researchers analysed 20 phenotypic traits pertinent to plant growth and reproduction. The findings revealed that in 11 of the 12 lines, 18 (90%) of the 20 traits exhibited stable inheritance across two apomictic generations cultivated concurrently in a randomized design, with only one line demonstrating instability (Sailer *et al*., 2016). A similar phenomenon was observed in a study where 2 out of 18 lines displayed unstable induction rates, with high rates in T_1_ and T_3_ generations and low rates in T_2_ and T_4_ generations (Dan *et al*., 2024). In our study, the L47‐4T_1_ generation displayed a high induction rate, reaching 83.8%, with a proportion of non‐fluorescent seeds of 100%; however, in the T_2_ generation, the induction rate dropped to 9.7%, and the proportion of non‐fluorescent seeds decreased to 2.8%. Based on these findings, we hypothesize that the L47‐4 strain may have undergone genetic instability. Due to the application of the pollen‐specific gene switch system, we successfully isolated 80.2% (*n* = 121) diploid plants from the non‐fluorescent seeds of the L47‐4T_2_ generation. Consequently, in light of this pronounced genetic instability, the utilization of the pollen‐specific gene switch system in apomixis research is particularly crucial and imperative.

**Figure 5:**
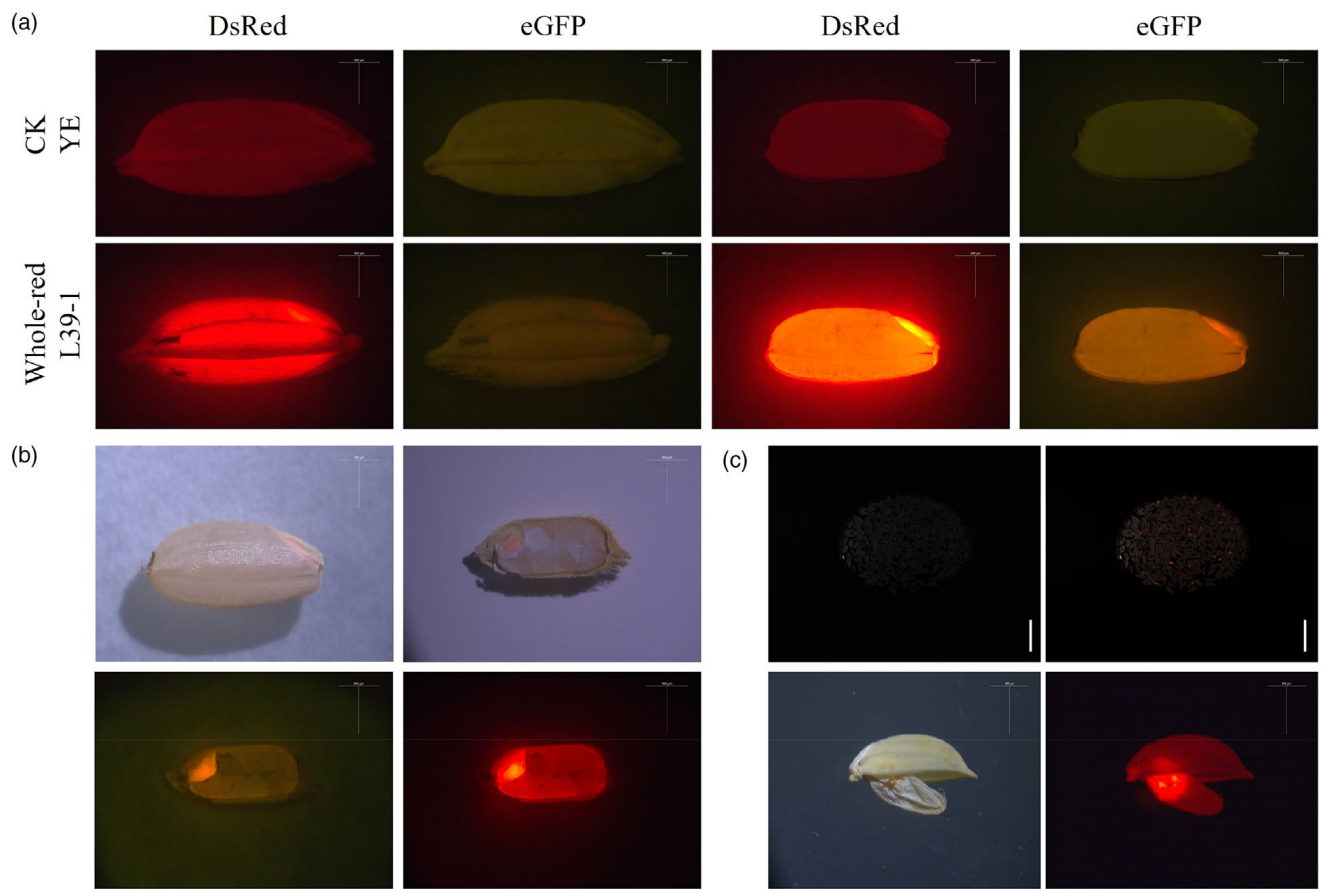
Fluorescence characteristics of L39‐1 seeds. (a) L39‐1 seeds observed under red fluorescence and green fluorescence illumination in a stereomicroscope. Scale bars, 500 μm. (b) Bright‐field observation of L39‐1 seeds after removing the glumes and cross‐sectional fluorescence observation of the L39‐1 seeds. Scale bars, 500 μm. (c) The population and individuals of shrunken grains under bright‐field and red fluorescence field conditions. Scale bars, 5 cm for shrunken grains; Scale bars, 500 μm for shrunken grain.

In our system, we have identified certain limitations, including that the sorting accuracy has not yet reached the perfect level of precision, and the fluorescent expression in some seeds does not consistently align with our expectations. The suboptimal accuracy in sorting may be due to the effectiveness of the *Cre/Loxp* recombinase system's deletion process, while the variable fluorescent expression is likely a result of the limited specificity of the pollen‐specific promoter. This is particularly noticeable in line L39‐1, which shows a unique fluorescence expression pattern compared with lines L47‐4 and L151‐1. In line L39‐1, both the seed embryo and endosperm exhibit strong red fluorescence when illuminated with red light (Figure 5a); under green fluorescence illumination, it assumes a yellow hue (Figure 5a). Once the glume is removed, the red fluorescence of the embryo is readily visible to the naked eye (Figure 5b). After cross‐sectioning the seeds, the embryo region was the only area to display fluorescence visible to the naked eye under bright‐field conditions. In the green fluorescence field, the embryo region exhibited yellow fluorescence, whereas in the red fluorescence field, it demonstrated intense red fluorescence, and the endosperm also exhibited red fluorescence (Figure 5b). Genomic DNA was extracted from the leaves of the L39‐1T_1_ generation (*n* = 23), and PCR amplification was conducted using primers designed to detect the deletion band. The molecular detection results of the L39‐1T_1_ generation indicated that only the deleted bands were present in all lines (Figure S2), suggesting that the green fluorescence *eGFP* fragment had been completely excised in the L39‐1T_1_ generation. Given that both *DsRed* and *eGFP* showed positive results at the three‐leaf stage in the L39‐1T_0_ generation, it is inferred that the insufficient specificity of the pollen‐specific promoter led to the expression of the recombinase after the three‐leaf stage, triggering the premature activation of the gene switch in the plants and deleting the *eGFP* green fluorescence gene between the recognition sites. Therefore, the fragments located between the recognition sites in sperm and egg cells were completely excised, resulting in the pronounced expression of red fluorescence. To address these issues, our future strategies include replacing the current pollen‐specific promoter or integrating more suitable endogenous pollen‐specific promoters into the rice genome and altering the intron insertion sites. These modifications are aimed at enhancing the specificity of the system and ensuring that fluorescent expression is confined to the intended tissues, thereby improving the overall efficiency and accuracy of our sorting process.

Within the shrunken grains of L39‐1, the embryo was observed to exhibit red fluorescence (Figure 5c). In L39‐1, the *BBM1* gene was expressed in egg cells and promotes embryo formation but no endosperm development. Consequently, the failure of endosperm development results in seed abortion (Khanday *et al*., 2019). The integration of fluorescent genes into the system improves the visual detection of this process. To address the low seed‐setting rate, our future research will focus on enhancing the autonomous development of the endosperm. Subsequent studies will explore the integration of *BBM1* and genes associated with endosperm autonomous development to increase seed‐setting rates and achieve the goal of high induction rate, high seed‐setting rate and high sorting efficiency.

## Materials and methods

### Plant materials and growth environment

The plant materials used in this study comprised the indica‐japonica hybrid rice varieties Yongyou 2640 (YE) and Yongyou 4949 (YS). Transgenic plants from the T_0_, T_1_ and T_2_ generations, including YE and YS hybrids, were cultivated in transgenic greenhouses in Sanya City, Hainan Province and Changsha City, Hunan Province, China. The growth of these plants was managed following standard practices for transgenic crop cultivation.

### Plasmid construction

The plasmid construction process entailed the synthesis of a cassette under the control of the dual constitutive promoter *2Xp35S*, which comprised the red fluorescent gene *DsRed* and the terminator *T35S*. A corresponding cassette was also synthesized, containing the fusion recognition site *Loxp + FRT*, the green fluorescent gene *eGFP*, the terminator Nos and the *Loxp + FRT* sequence (designated as the ‘gene lock’). This ‘gene lock’ cassette was inserted between the *2Xp35S* promoter and the *DsRed* gene, thereby repressing the expression of the *DsRed* gene. Additionally, a cassette driven by the constitutive promoter *pUbi*, encoding the recombinase *Cre* and the terminator *PinII*, was synthesized (termed the ‘gene key’). These sequences were then subcloned into the vector backbone pC1300, which was employed in this study, yielding the gene switch vector: *2Xp35S‐Loxp + FRT‐eGFP‐Nos‐Loxp + FRT + DsRed‐T35S‐PinII‐Cre* ^*intron*^ *‐pUbi*, named p96C. The recombinase *FLP* was used in place of *Cre* in p96C to create the vector: *2Xp35S‐Loxp + FRT‐eGFP‐Nos‐Loxp + FRT + DsRed‐T35S‐PinII‐FLP* ^*intron*^ *‐pUbi*, named p97C. To reduce background expression of *Cre* and *FLP* in prokaryotes, a 190‐bp intron sequence was inserted into the recombinase coding sequence at the 5–6‐bp position (Guo *et al*., 1997; Ouedraogo *et al*., 2015).

The vector p96C was modified by replacing the constitutive promoter *pUbi* with the maize pollen‐specific promoter *pG47* (Xia *et al*., 2019), resulting in the vector *2Xp35S‐Loxp + FRT‐eGFP‐Nos‐Loxp + FRT + DsRed‐T35S‐PinII‐Cre* ^*intron*^ *‐pG47*, named p98C. Similarly, by substituting the constitutive promoter *pUbi* in p96C with the rice anther‐specific promoter *pv4* (Zhu *et al*., 2017), the vector *2Xp35S‐Loxp + FRT‐eGFP‐Nos‐Loxp + FRT + DsRed‐T35S‐PinII‐Cre* ^*intron*^ *‐pv4* was generated, named p99C.

We inserted the embryo‐specific promoter *Ole18* to regulate the specific expression of fluorescence in the seed embryo (Bu *et al*., 2022), combined with the pollen‐specific gene switch system, such that the clonal embryos produced by apomixis show no red fluorescence, while the zygotic embryos formed by pollination and fertilization exhibit red fluorescence. Based on this, a novel sequence, *Ole18‐Loxp + FRT‐2Xp35S*, was synthesized and then used to replace the *2Xp35S‐Loxp + FRT* sequence in p98C, yielding the vector *Ole18‐Loxp + FRT‐2Xp35S‐eGFP‐Nos‐Loxp + FRT‐DsRed‐T35S‐PinII‐Cre* ^*intron*^ *‐pG47*, designated as p100C. In a parallel manner, the *Ole18‐Loxp + FRT‐2Xp35S* sequence was substituted for the *2Xp35S‐Loxp + FRT* sequence in p99C, resulting in the vector *Ole18‐Loxp + FRT‐2Xp35S‐eGFP‐Nos‐Loxp + FRT‐DsRed‐T35S‐PinII‐Cre* ^*intron*^ *‐pv4*, named p106C. All sequences were synthesized and subcloned by Nanjing GenScript Biotech Co., Ltd. for this study.

### *Agrobacterium*‐mediated genetic transformation

The plasmids p100C and p106C were independently introduced into competent EHA105 cells, leading to the transformation of YE and YS, respectively. Subsequently, following this, callus induction and plant regeneration were conducted in accordance with the protocol outlined (Xia *et al*., 2019). Additionally, the pollen‐specific gene switch system (comprising p100C and p106C) was integrated with the apomictic vectors *‘sgMiMe’_pAtDD45:BBM1* (p94C) and *‘sgMiMe’_pAtMYB98 + pAtDD1 + pOsECA1‐like1:WUS_pAtDD45:BBM1* (p95C), which were developed in our laboratory, to enable co‐transformation.

### Callus establishment and biolistic transformation

Dehulled rice seeds were initially surface‐disinfected with 75% ethanol for 3–5 min, followed by sterilization in a 10% sodium hypochlorite solution (Tianjin Damao) for 20–30 min. Afterwards, the seeds were washed with sterile water four to five times. The disinfected seeds were then inoculated onto NB medium and incubated at 26–28 °C in the dark to facilitate callus initiation. After 1 week of culture, the delicate yellow YE callus was excised into 0.4 cm squares and evenly distributed within a petri dish lined with a high‐osmotic medium. The callus was then incubated in a dark incubator at 28 °C for 4 h.

For nuclear transformation, 60 mg of gold powder with a particle size of 1 μm, and for chloroplast transformation, 60 mg of gold powder with a particle size of 0.6 μm, were each weighed into a silicon‐treated 1.5 mL centrifuge tube. The powder was supplemented with 1 mL of absolute ethanol and vortexed for 1 min at a low speed. The mixture was then centrifuged at 500 rpm for 25 s, and the supernatant was discarded. This centrifugation step was repeated once. The gold powder was subsequently resuspended in 1 mL of sterile water for immediate use. A 100 μL aliquot of the gold suspension was transferred to a 1.5 mL centrifuge tube, to which 5 μL of a 1 μg/μL plasmid solution was added. The mixture was vortexed, and during this process, 40 μL of 0.1 mol/L spermine and 100 μL of 2.5 mol/L CaCl_2_ solution were sequentially added. After vortexing for 3 min, the mixture was allowed to stand for 2 min before being centrifuged again at 500 rpm for 25 s. If the liquid appeared cloudy, the centrifugation step was repeated, and the supernatant was discarded. The gold powder was resuspended in 250 μL of absolute ethanol for subsequent rounds of biolistic bombardment for a total of 10 rounds. For each round, 10 μL of DNA microsphere suspension was applied evenly onto the flight membrane. The vacuum was set to 700 MPa, maintaining a 1 cm gap between the flight membrane and the carrier membrane, and a 5 cm distance between the carrier membrane and the callus. Each dish was bombarded twice, with the material rotated by 90° for the second round. Following bombardment, the dishes were incubated in a dark environment at room temperature for 16 h before being transferred to NB medium, ensuring a 1 cm spacing between each callus for subsequent observation.

### Pollen biolistic transformation

Pollen grains from the YE line were harvested from a liquid culture medium, after which the culture medium was removed by filtration through a sterile filter paper formed into a funnel (Wang and Jiang, 2011). The filtered pollen was then evenly distributed on the filter paper and subsequently transferred to a solid culture medium for the biolistic bombardment process.

### Confirmation of transgenic plant status

Genomic DNA was isolated from the leaves of both transgenic and control plants employing a rapid extraction protocol (Zhan, 2002). Polymerase chain reaction (PCR) amplification of the *OSD1*, *PAIR1* and *REC8* genes was carried out using the Novozymes 2X Taq PCR Mix. The PCR products were subjected to Sanger sequencing, and the resulting sequences were analysed with Sequencer 5.4.6 software (Tsingke Biotechnology Co., Ltd., Beijing, China). Similarly, PCR amplification of the *BBM1*, *eGFP* and *DsRed* genes was performed using the same PCR mix. The amplified PCR fragments were compared and analysed using DNAMAN 6.0 software (refer to the Table S5 for details).

### Fluorescence observation

The fluorescence phenotypes of seeds in the field were observed and documented with a portable fluorescence visualization device, the DFP‐1. The fluorescence in callus tissues and transgenic seeds was examined and recorded using a fully automated fluorescence stereomicroscope (MZ16FA; Leica, Wetzlar, Germany), fitted with an X10 objective lens and an X70 eyepiece for callus observations, and an X10 objective lens with an X7 eyepiece for the examination of transgenic seeds. Additionally, the fluorescence of shrunken grains was monitored and documented using a plant live molecular imaging system (NEWTON7.0 Bio plus; VILBER, Pairs, France).

### Flow cytometry analysis

The nuclear DNA content was measured following the protocol provided with the CyStain™ PI Absolute P reagent kit. A flow cytometer (FongCyte C2060; Beijing Challen Biotechnology Co., Ltd., Beijing, China) was utilized to assess the ploidy levels in both transgenic lines and control plants.

### Identification of deleted sequences

Primers were designed to specifically amplify the deleted fragment at the *Loxp + FRT* fusion site. PCR was conducted to distinguish between the undeleted and deleted DNA bands using the Novozymes 2X Taq PCR Mix (refer to Table S5 for primer details). The PCR products were subjected to Sanger sequencing, and the resulting sequences were analysed with Sequencer 5.4.6 software (Tsingke Biotechnology Co., Ltd.).

### Agronomic characterization

The evaluated agronomic traits included panicle length, effective panicle number and seed‐setting rate.

## Conflict of interest

The authors declare that they have no conflict of interest.

## Authors' contribution

M.C., Y.Z. and Y.X. designed the experiments and analysed the data. Y.Z. and Y.W. performed the most experiments, with the assistance of M.C., Y.X., S.L., X.Z., S.X. and Q.L. Y.Z., Y.X., S.X., Q.L. and M.C. wrote the paper. All authors have read and commented on the paper.

## Supporting information


**Figure S1** *MiMe* mutation analysis.
**Figure S2** Phenotypic characterization of the T_1_ generation of hybrid rice transformation offspring.
**Figure S3** PCR analysis of seeds with varying fluorescence expressions.
**Figure S4** Sequencing analysis results.
**Table S1** Quantitative analysis of red and green gluorescence bectors.
**Table S2** Analysis of co‐transformation efficiency and detection of transformed materials.
**Table S3** Quantitative analysis of non‐fluorescent seeds in T_2_ generation of L47‐4 and L151‐1.
**Table S4** Flow cytometry analysis and field ploidy assessment of L47‐4 and L151‐1.
**Table S5** PCR and sequencing primers sets.

## Data Availability

The data that supports the findings of this study are available in the supplementary material of this article.

## References

[pbi70031-bib-0001] Bu, X. , Xia, Y. , Zhan, Y. , Yu, M. , Dan, J. , Tang, N. and Cao, M. (2022) Visualizing the function of embryo‐specific promoters using dual fluorescent reporter genes. Mol. Plant Breed. 11, 3587–3594.

[pbi70031-bib-0002] Chen, H. , Luo, J. , Zheng, P. , Zhang, X. , Zhang, C. , Li, X. , Wang, M. *et al*. (2017) Application of Cre‐lox gene switch to limit the Cry expression in rice green tissues. Sci. Rep. 7, 14505. 29109405 10.1038/s41598-017-14679-0 PMC5673937

[pbi70031-bib-0003] Dan, J. , Xia, Y. , Wang, Y. , Zhan, Y. , Tian, J. , Tang, N. , Deng, H. *et al*. (2024) One‐line hybrid rice with high‐efficiency synthetic apomixis and near‐normal fertility. Plant Cell Rep. 43, 79. 38400858 10.1007/s00299-024-03154-6 PMC10894110

[pbi70031-bib-0004] Guo, F. , Gopaul, D.N. and Van Duyne, G.D. (1997) Structure of Cre recombinase complexed with DNA in a site‐specific recombination synapse. Nature, 389, 40–46. 9288963 10.1038/37925

[pbi70031-bib-0019] Hu, T. (2013) A Novel Safe Transgenic Rice Breeding Technology Based on Molecular Deletion Strategy. Fuzhou, China: Fujian Agriculture and Forestry University.

[pbi70031-bib-0005] Huang, Y. , Meng, X. , Rao, Y. , Xie, Y. , Sun, T. , Chen, W. , Wei, X. *et al*. (2024) OsWUS‐driven synthetic apomixis in hybrid rice. Plant Commun. 6, 101136. 39305015 10.1016/j.xplc.2024.101136 PMC11783873

[pbi70031-bib-0006] Khanday, I. , Skinner, D.J. , Yang, B. , Mercier, R. and Sundaresan, V. (2019) A male‐expressed rice embryogenic trigger redirected for asexual propagation through seeds. Nature, 565, 91–95. 30542157 10.1038/s41586-018-0785-8

[pbi70031-bib-0007] Khanday, I. , Santos‐Medellín, C. and Sundaresan, V. (2023) Somatic embryo initiation by rice BABY BOOM1 involves activation of zygote‐expressed auxin biosynthesis genes. New Phytol. 238, 673–687. 36707918 10.1111/nph.18774

[pbi70031-bib-0008] Luo, H. and Kausch, A.P. (2002a) Application of FLP/FRT site‐specific DNA recombination system in plants. In Genetic Engineering: Principles and Methods, Vol. 24 ( Setlow, J.K. , ed), pp. 1–16. New York, NY: Springer. 10.1007/978-1-4615-0721-5_1 12416298

[pbi70031-bib-0009] Luo, H. and Kausch, A.P. (2002b) Application of FLP/FRT site‐specific DNA recombination system in plants. Genet. Eng. (N Y), 24, 1–16. 12416298 10.1007/978-1-4615-0721-5_1

[pbi70031-bib-0010] Luo, K. , Duan, H. , Zhao, D. , Zheng, X. , Deng, W. , Chen, Y. , Stewart, C.N., Jr. *et al*. (2007) ‘GM‐gene‐deletor’: fused loxP‐FRT recognition sequences dramatically improve the efficiency of FLP or CRE recombinase on transgene excision from pollen and seed of tobacco plants. Plant Biotechnol. J. 2, 263–274. 10.1111/j.1467-7652.2006.00237.x 17309681

[pbi70031-bib-0011] Nandy, S. and Srivastava, V. (2011) Site‐specific gene integration in rice genome mediated by the FLP–FRT recombination system. Plant Biotechnol. J. 9, 713–721. 21083801 10.1111/j.1467-7652.2010.00577.x

[pbi70031-bib-0012] Nguyen, L.D. , Underwood, J.L. , Nandy, S. , Akbudak, M.A. and Srivastava, V. (2014) Strong activity of FLPe recombinase in rice plants does not correlate with the transmission of the recombined locus to the progeny. Plant Biotechnol. Rep. 8, 455–462.

[pbi70031-bib-0013] Ouedraogo, J.P. , Arentshorst, M. , Nikolaev, I. , Barends, S. and Ram, A.F. (2015) I‐Sce I‐mediated double‐strand DNA breaks stimulate efficient gene targeting in the industrial fungus Trichoderma reesei. Appl. Microbiol. Biotechnol. 99, 10083–10095. 26272087 10.1007/s00253-015-6829-1 PMC4643118

[pbi70031-bib-0014] Rao, M.R. , Moon, H.S. , Schenk, T.M. , Becker, D. , Mazarei, M. and Stewart, C.N., Jr. (2010) FLP/FRT recombination from yeast: application of a two gene cassette scheme as an inducible system in plants. Sensors, 10, 8526–8535. 22163670 10.3390/s100908526 PMC3231192

[pbi70031-bib-0015] Sailer, C. , Schmid, B. and Grossniklaus, U. (2016) Apomixis allows the transgenerational fixation of phenotypes in hybrid plants. Curr. Biol. 26, 331–337. 26832437 10.1016/j.cub.2015.12.045

[pbi70031-bib-0016] Song, M. , Wang, W. , Ji, C. , Li, S. , Liu, W. , Hu, X. , Feng, A. *et al*. (2024) Simultaneous production of high‐frequency synthetic apomixis with high fertility and improved agronomic traits in hybrid rice. Mol. Plant, 17, 4–7. 37990497 10.1016/j.molp.2023.11.007

[pbi70031-bib-0017] Srivastava, V. and Nicholson, S.J. (2006) Cre/lox technologies for plant transformation. CABI Rev., 34, 12.

[pbi70031-bib-0018] Stachowski, K. , Norris, A.S. , Potter, D. , Wysocki, V.H. and Foster, M.P. (2022) Mechanisms of Cre recombinase synaptic complex assembly and activation illuminated by Cryo‐EM. Nucleic Acids Res. 50, 1753–1769. 35104890 10.1093/nar/gkac032 PMC8860596

[pbi70031-bib-0020] Vernet, A. , Meynard, D. , Lian, Q. , Mieulet, D. , Gibert, O. , Bissah, M. , Rivallan, R. *et al*. (2022) High‐frequency synthetic apomixis in hybrid rice. Nat. Commun. 13, 7963. 36575169 10.1038/s41467-022-35679-3 PMC9794695

[pbi70031-bib-0021] Wang, H. and Jiang, L. (2011) Transient expression and analysis of fluorescent reporter proteins in plant pollen tubes. Nat. Protoc. 6, 419–426. 21412270 10.1038/nprot.2011.309

[pbi70031-bib-0022] Wang, C. , Liu, Q. , Shen, Y. , Hua, Y. , Wang, J. , Lin, J. , Wu, M. *et al*. (2019) Clonal seeds from hybrid rice by simultaneous genome engineering of meiosis and fertilization genes. Nat. Biotechnol. 37, 283–286. 30610223 10.1038/s41587-018-0003-0

[pbi70031-bib-0023] Wei, X. , Liu, C. , Chen, X. , Lu, H. , Wang, J. , Yang, S. and Wang, K. (2023) Synthetic apomixis with normal hybrid rice seed production. Mol. Plant, 16, 489–492. 36609144 10.1016/j.molp.2023.01.005

[pbi70031-bib-0024] Wen, M. (2015) Analysis of Deletion Efficiency in Transgenic Arabidopsis Hybrid Offspring Using the Cre/Loxp System Based on Protein Split Technology. El Paso, TX: Southwest University.

[pbi70031-bib-0025] Xia, Y. , Tang, N. , Hu, Y. , Li, D. , Li, S. , Bu, X. , Yu, M. *et al*. (2019) A method for mechanized hybrid rice seed production using female sterile rice. Rice, 12, 1–10. 31140005 10.1186/s12284-019-0296-8 PMC6538728

[pbi70031-bib-0026] Yuan, L. (1987) Strategic considerations for the breeding of hybrid rice. Hybrid Rice, 1, 1–3.

[pbi70031-bib-0027] Zhan, Q. (2002) Research on the purity identification of hybrid rice seeds using microsatellite DNA markers. Hybrid Rice, 5, 48–52.

[pbi70031-bib-0028] Zhao, Y. , Zhang, X. , Guo, L. and Qian, Q. (2011) Efficient deletion of marker genes in transgenic rice at the transformed cell level using the Cre/Loxp system. Chin. J. Rice Sci. 25, 580–586.

[pbi70031-bib-0029] Zhu, Q. , Yu, S. , Zeng, D. , Liu, H. , Wang, H. , Yang, Z. , Xie, X. *et al*. (2017) Development of “purple endosperm rice” by engineering anthocyanin biosynthesis in the endosperm with a high‐efficiency transgene stacking system. Mol. Plant, 10, 918–929. 28666688 10.1016/j.molp.2017.05.008

