# Supplementary material for: Efficient clonal seeds sorting for apomictic hybrid rice using a pollen‐specific gene switch system
Source: Plant Biotechnol J. 2025 Mar 19;23(6):2266–75. doi: 10.1111/pbi.70031 (PMC12120867; doi:10.1111/pbi.70031)
Supplement: Supplementary file 1 — Figure S1 MiMe mutation analysis. Figure S2 Phenotypic characterization of the T1 generation of hybrid rice transformation offspring. Figure S3 PCR analysis of seeds with varying fluorescence expressions. Figure S4 Sequencing analysis results. Table S1 Quantitative analysis of red and green gluorescence bectors. Table S2 Analysis of co‐transformation efficiency and detection of transformed materials. Table S3 Quantitative analysis of non‐fluorescent seeds in T2 generation of L47‐4 and L151‐1. Table S4 Flow cytometry analysis and field ploidy assessment of L47‐4 and L151‐1. Table S5 PCR and sequencing primers sets. [file PBI-23-2266-s001.doc]

**Supplemental Table 1** Quantitative analysis of red and green gluorescence bectors.

| Plasmid Content（ng） | Vector Name | Plate 1 | | Plate 2 | | Plate 3 | |
| --- | --- | --- | --- | --- | --- | --- | --- |
|  |  | eGFP particles | DsRed particles | eGFP particles | DsRed particles | eGFP particles | DsRed particles |
| 2 | p96C | 0 | 33 | 11 | 50 | 16 | 39 |
|  | p97C | 4 | 11 | 23 | 20 | 9 | 18 |
| 5 | p96C | 19 | 79 | 7 | 77 | 22 | 296 |
|  | p97C | 12 | 20 | 26 | 28 | 31 | 9 |

**Supplemental Table 2** Analysis of co-transformation efficiency and detection of transformed materials.

| Vector Transformation Status | Number of Plants Obtained | Number of BBM1 positive | Number of BBM1 and DsRed positive | Number of *MiMe* mutation and DsRed positive | Co-transformation Efficiency |
| --- | --- | --- | --- | --- | --- |
| p100C-p94C transformed to YE T0 | 56 | 26 | 16 | 11 | 19.6% |
| p100C-p95C transformed to YS T0 | 65 | 40 | 4 | 3 | 4.6% |
| p106C-p94C transformed to YS T0 | 64 | 29 | 2 | 2 | 3.1% |
| p106C-p95C transformed to YE T0 | 57 | 25 | 3 | 2 | 3.5% |

**Supplemental Table 3** Quantitative analysis of non-fluorescent seeds in T_2_ generation of L47-4 and L151-1.

| Plants | Serial number | Number of non-fluorescen | Number of filled grain | Ratio of non-fluorescen |
| --- | --- | --- | --- | --- |
| L47-4 | 1 | 24 | 935 | 2.6% |
|  | 2 | 19 | 741 | 2.6% |
|  | 3 | 17 | 669 | 2.5% |
|  | 4 | 20 | 830 | 2.4% |
|  | 5 | 40 | 1084 | 3.7% |
|  | 6 | 22 | 776 | 2.8% |
|  | 7 | 23 | 877 | 2.6% |
|  | 8 | 22 | 768 | 2.9% |
| Total |  | 187 | 6680 | 2.8% |
| L151-1 | 1 | 133 | 1270 | 10.5% |
|  | 2 | 89 | 998 | 8.9% |
|  | 3 | 139 | 1312 | 10.6% |
|  | 4 | 97 | 1031 | 9.4% |
|  | 5 | 156 | 1408 | 11.1% |
|  | 6 | 148 | 1439 | 10.3% |
|  | 7 | 130 | 1222 | 10.6% |
|  | 8 | 126 | 1108 | 11.4% |
| Total |  | 1018 | 9788 | 10.4% |

Eight T_1_ progeny of each L47-4 and L151-1 plants were randomly selected as reference.

**Supplemental Table 4** Flow cytometry analysis and field ploidy assessment of L47-4 and L151-1.

| Generation | Transgenic line# | Number of  diploids | Number of  tetraploids | Total plants |
| --- | --- | --- | --- | --- |
| T1 | L47-4 | 57 | 11 | 68 |
| T2 | L47-4-1 | 13 | 5 | 18 |
|  | L47-4-2 | 10 | 0 | 10 |
|  | L47-4-3 | 9 | 1 | 10 |
|  | L47-4-4 | 10 | 4 | 14 |
|  | L47-4-5 | 17 | 5 | 22 |
|  | L47-4-6 | 13 | 1 | 14 |
|  | L47-4-7 | 13 | 3 | 16 |
|  | L47-4-8 | 12 | 5 | 17 |
| T1 | L151-1 | 64 | 8 | 72 |
| T2 | L151-1-1 | 21 | 4 | 25 |
|  | L151-1-2 | 17 | 3 | 20 |
|  | L151-1-3 | 22 | 3 | 25 |
|  | L151-1-4 | 16 | 5 | 21 |
|  | L151-1-5 | 23 | 2 | 25 |
|  | L151-1-6 | 22 | 3 | 25 |
|  | L151-1-7 | 23 | 0 | 23 |
|  | L151-1-8 | 20 | 3 | 23 |

**Supplemental Table 5 PCR and sequencing primers sets.**

| Primer name | Primer sequence (5' - 3') | Notes |
| --- | --- | --- |
| OSD1-F | GGGATTCGTTGGTTCGTGTT | Mutation detection of *OSD1.* |
| OSD1-R | TTGCGATAAGCAGAAAGAAATG |
| PAIR1-F | CGAAGGAGAAGGCTACGGC | Mutation detection of *PAIR1.* |
| PAIR1-R | CAGGGACAGGAGTGAGTGGAA |
| REC8-F | GCGACGCTTCACTCGAAGATCA | Mutation detection of *REC8.* |
| REC8-R | CGCCATGCCTCGTTGATCTCAA |
| BBM1-L | CAGCATGGAAGGTGGCAAGCAAG | Mutation detection of *BBM1.* |
| BBM1-R | GCAAGAAGCGAACCCTCGGAAAG |
| eGFP-F | GGCAAGCTGACCCTGAAGTT | Mutation detection of *eGFP.* |
| eGFP-R | TCTCGTTGGGGTCTTTGCTC |
| DsRed-L | GAAGCTGAAGGTGACGAAGG | Mutation detection of *DsRed.* |
| DsRed-R | GCTCCACGATGGTGTAGTCC |
| Loxp-L | CTCCTCCTCCCTACGTGTCA | Amplification and sequencing of the non-deleted fragment and the fragment after deletion. |
| Loxp-R | CCCTTCGTCACCTTCAGCTT |
| JC-R | CGGCGGCGGTCACGAACTCCAGCAG | Amplification and sequencing of the intermediate fragment. |


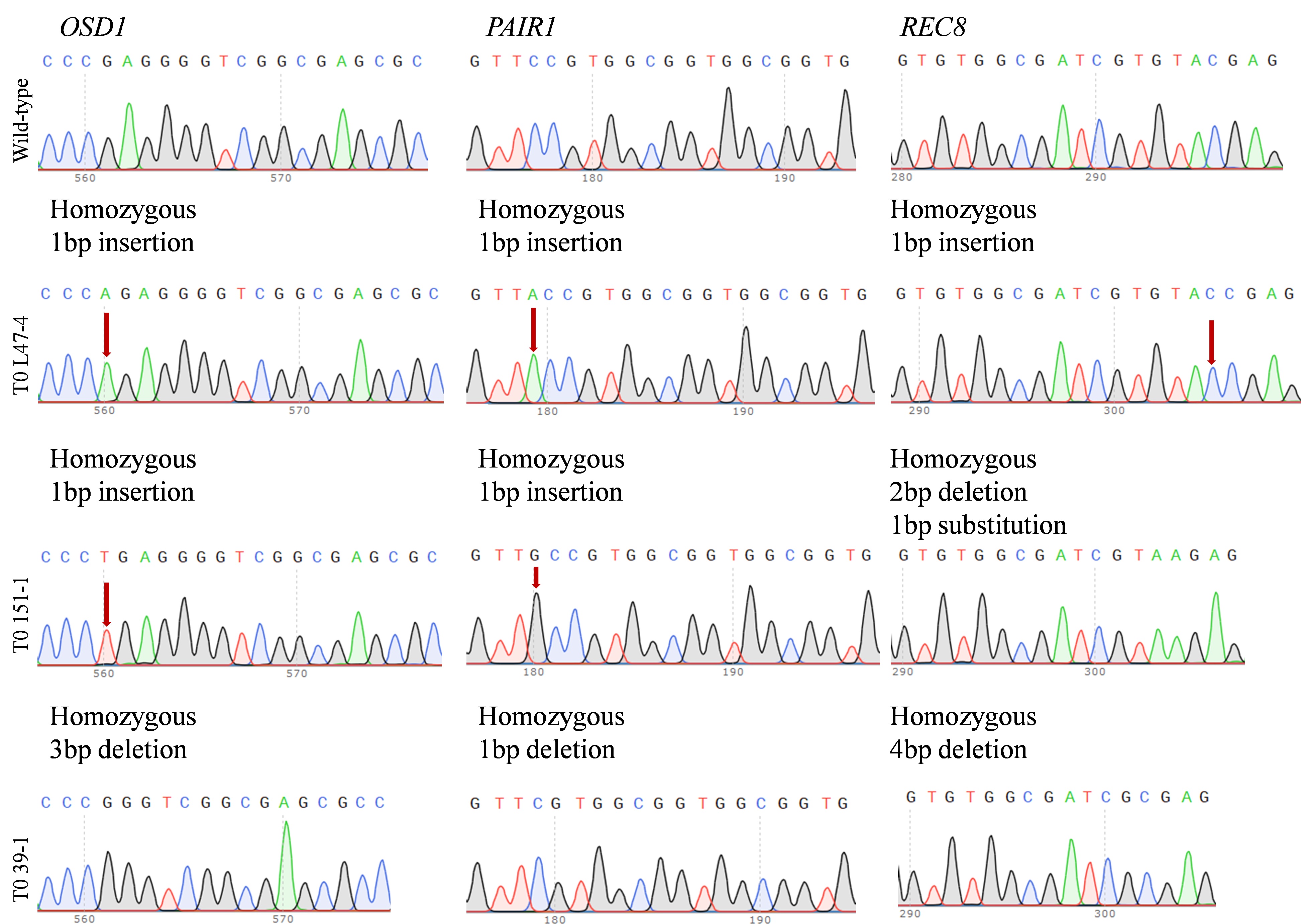


**Supplemental Figure 1** *MiMe* mutation analysis. Chromatographic profiles of *MiMe* gene mutation sites in wild-type and T_0_ PSGS mother plants. Red arrows indicate mutation sites. *OSD1*、*PAIR1* and *REC8* are homozygous in L47-4、L151-1 and L39-1.


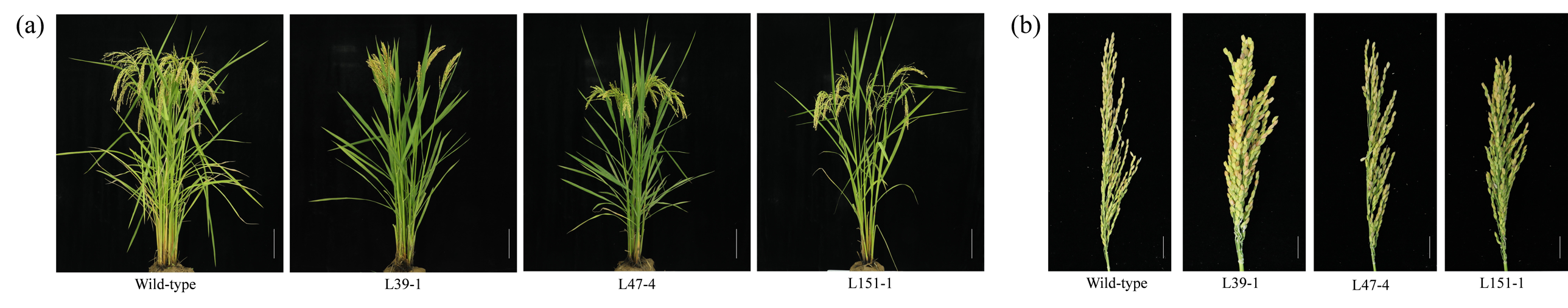


**Supplemental Figure 2** Phenotypic characterization of the T_1_ generation of hybrid rice transformation offspring. (a) Morphological comparison of L39-1, L47-4, and L151-1 with the wild type. Scale bar, 10 cm. (b) Panicle trait comparison between T_1_ diploid clone plants and the wild type. Scale bar, 2 cm.


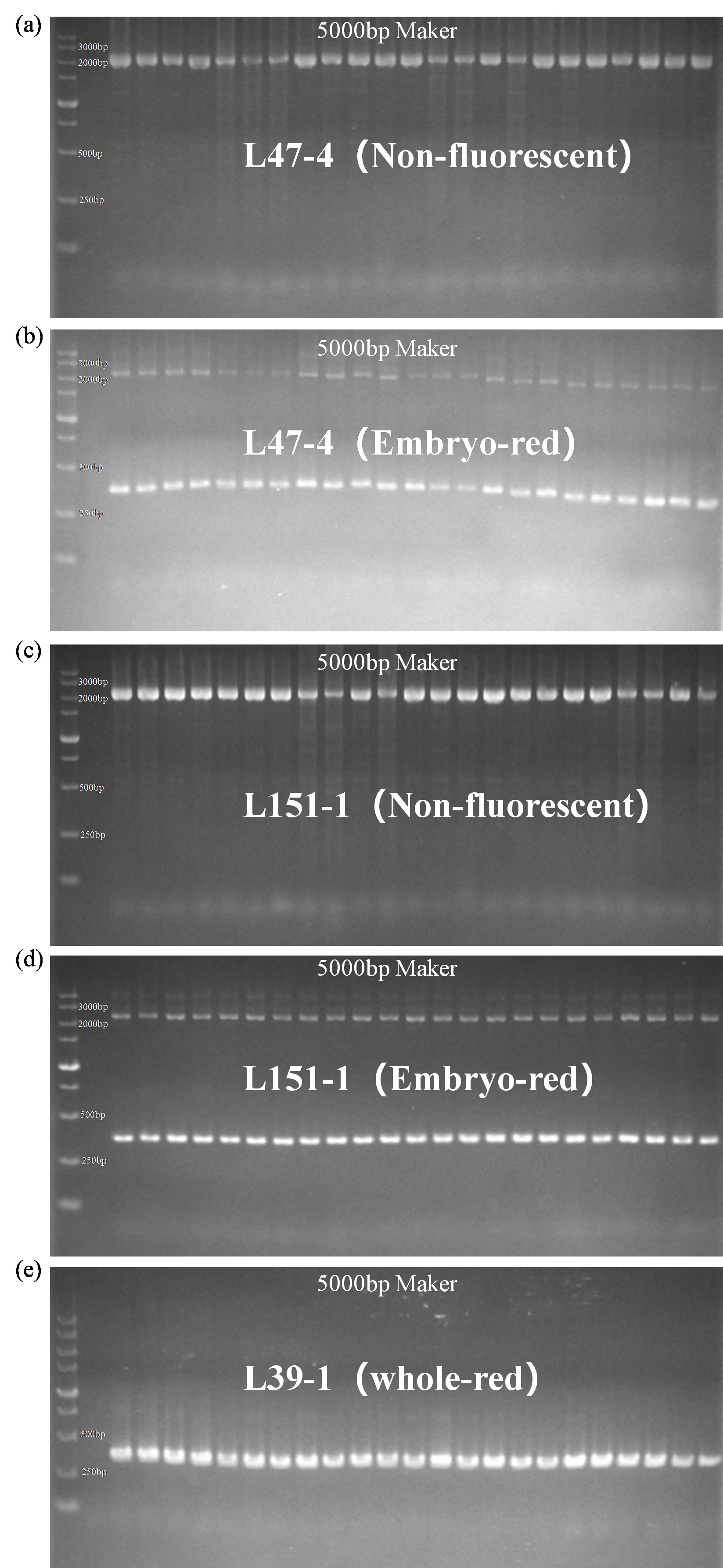


**Supplemental Figure3** PCR analysis of seeds with varying fluorescence expressions. (a) The first sample in row represents hybrid rice YY2640, followed by 23 randomly selected L47-4 non-fluorescent T_1_ diploids plants. (b) The first sample in row represents hybrid rice YY2640, followed by 23 randomly selected L47-4 embryo-red T_1_ plants. (c) The first sample in row is hybrid rice YY2640, the other 23 were randomly selected L47-4 non-fluorescent T_1_ diploids plants. (d) The first sample in row is hybrid rice YY2640, the other 23 were randomly selected L47-4 embryo-red T_1_ plants. (e) The first sample in row is hybrid rice YY2640, the other 23 were randomly selected L39-1 “whole-red” T_1_ plants.


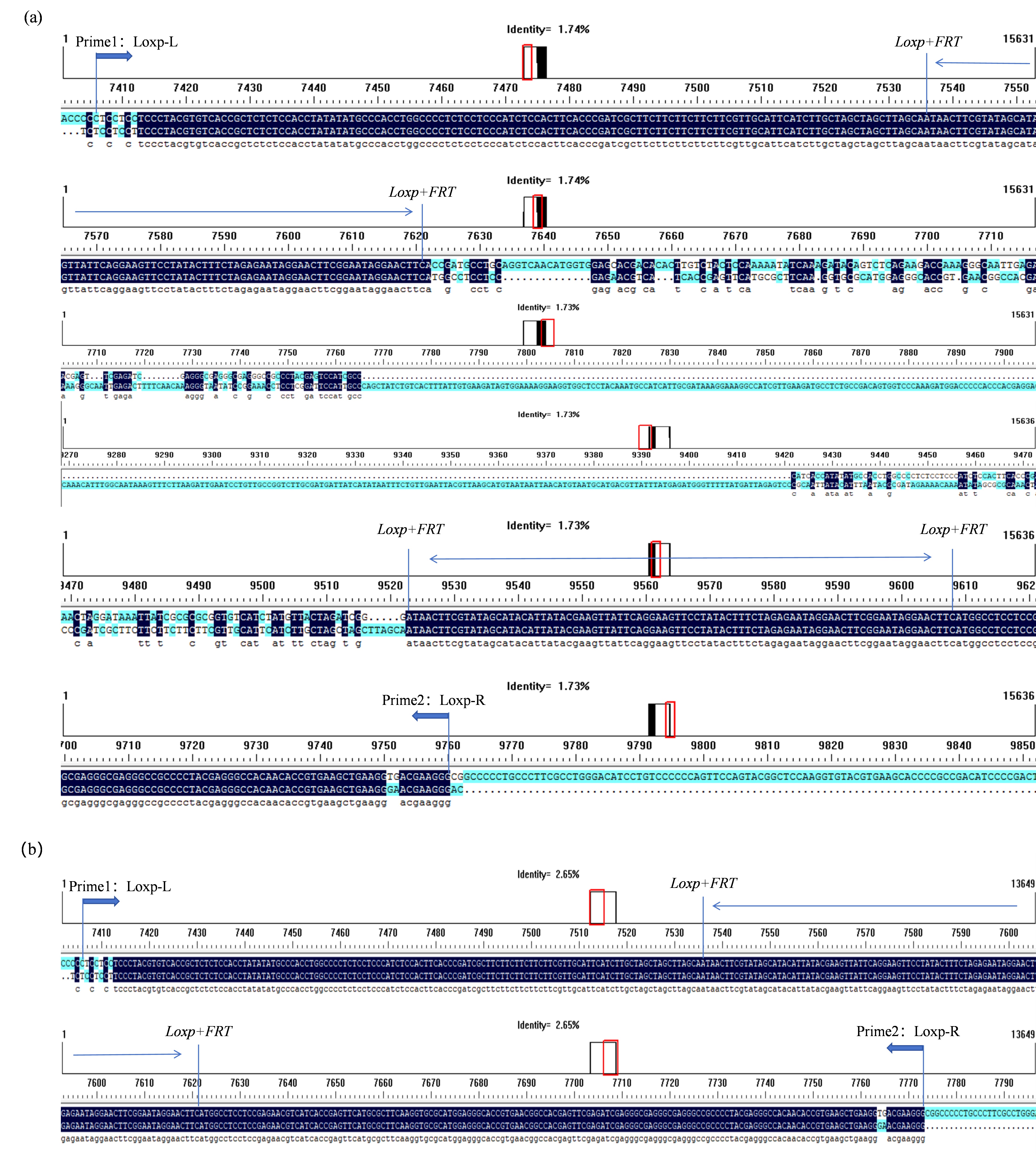


**Supplemental Figure4** Sequencing analysis results. (a) Alignment of the deleted fragment with the original sequence. The figure annotates the positions of primer1: Loxp-L, primer2: Loxp-R, and the two fusion recognition sites *Loxp+FRT*. (b) Comparison of the spliced deleted fragment with the simulated original sequence after deletion.
